# Are remote psychotherapy/remediation efforts accessible and feasible in patients with schizophrenia? A narrative review

**DOI:** 10.1186/s41983-022-00574-7

**Published:** 2022-11-18

**Authors:** Reetobaan Datta, Rashmi Vishwanath, Sonia Shenoy

**Affiliations:** 1 Department of Psychiatry, Kasturba Medical College, Manipal, India; 2 Manipal Academy of Higher Education, Manipal, Karnataka 576104 India

**Keywords:** Schizophrenia, Remote cognitive remediation, Psychotherapy, Cognitive therapy, Remote interventions

## Abstract

**Background:**

Cognitive remediation (CR) therapy provides an effective way to improve cognitive impairments in schizophrenia. With the advent of telehealth services, especially during COVID 19 pandemic, a suitable alternative can be found in computer and cell phone-based mental health interventions. Previous studies have proven that remote mental health interventions have by and large been successful. Remote psychotherapy/CR services can now be accessed through smartphone apps, iPads, laptops and wearable devices. This has the advantage of reaching a wider population in resource-limited settings. The lack of access to technology, difficulty in using these online interventions and lack of privacy provide impediments to the delivery of care through these online platforms. Further, as some previous studies have shown, there may be a high rate of dropout in people using remote mental health resources. We aim to look at the factors, which influence the accessibility of remote mental health interventions in schizophrenia. Additionally, we test the feasibility of these interventions and look at how they compare and the potential they hold for implementation in future clinical settings.

**Results:**

We found remote cognitive remediation to be both accessible and feasible. Concerning features, however, are the high attrition rates and the concentration of the studies in Western populations.

**Conclusions:**

Remote interventions are a viable alternative to in-person psychotherapy when in-person resources may not always be present. They are efficacious in improving health outcomes among patients with schizophrenia. Further research into the widespread implementation of remote CR will be beneficial in informing clinical decision-making.

## Introduction

Schizophrenia is a severe mental illness that is characterized by delusions and hallucinations, negative symptoms and cognitive deficits that cause lasting disability and impairment. It has been noted that about 75% of patients with schizophrenia have some form of neurocognitive deficits [1]. Cognitive remediation (CR) has been successful in reducing these deficits and help patients affect functionality in their daily lives [2]. During public health crises like the COVID-19 pandemic, light has been shed on the impediments of seeking therapy, such as the high cost of therapy and long distance of travel [3].

Cognitive remediation efforts have shown improvements in cognition, symptom control and psychosocial functioning [2]. The overall effects of cognitive remediation in improving cognition are present across all six of the domains with variable levels of success [4–9]. Evidence supports the integration of cognitive training alongside psychosocial and vocation rehabilitation programs [4, 6]. Cognitive training has further been supported by neurobiological studies, which have shown an increase in grey matter preservation in patients receiving CR [10].

Remote mental health interventions provide an effective way of offering help to patients [11]. Cognitive behavior therapy can be delivered through or enhanced by remote applications in multiple ways such as tele-psychiatry [12], smartphone applications [13], wearable devices [14], texting [13], web-based platforms [15] and virtual reality [16]. E-mental health interventions can facilitate expanded health care reach, improved clinical decision-making and sustained clinical contact during emergencies [17].

Guidelines for the use of internet interventions in psychosis [18, 19] recommend that remote interventions be developed in accordance with stakeholder input and online-based interventions augment rather than replace existing models of care. In addition, it recommends that technology be integrated with evidence based therapy and professional support to promote acceptance and effectiveness.

However, remote interventions especially psychotherapy and remediation are still under researched. It is unclear if it is accessible and feasible in patients with schizophrenia. This review aims to assess the existing literature specifically looking at feasibility, accessibility and challenges in remote psychotherapy and remote cognitive remediation in patients with schizophrenia.

## Accessibility of remote cognitive remediation

Differences in age, gender and level of education have not been found to affect the efficacy of traditional cognitive remediation therapy (CRT) [9]. However, when it comes to remote interventions in this regard, there have been differences. The accessibility reduces across an older age, women and non-Caucasian racial groups [20, 21]. This is concerning since minority and vulnerable groups especially lack the critical interventions needed. There is a need to address the mental health issues that are prevalent among vulnerable groups and improve access to remote interventions [22].

Medalia and colleagues [23] noted the lack of access to technology as a key barrier in the access to remote CR. In a study done to assess the use of telehealth services, it was found that the uninsured (compared with private insurance: OR = 0.4, 95% CI 0.2–0.8), and those with limited broadband coverage in the community (OR = 0.5, 95% CI 0.3–0.8) used remote health interventions to a lesser degree [24]. Having greater outreach efforts and educating patients on tasks such as installing telehealth applications and the usage of these avenues is required to improve the usage of such systems [25].

Baseline cognition has been found to have a positive association with the usage of CR [9]. However, there is little research on remote interventions and whether accessibility, continuation or discontinuation of therapy is dependent on the baseline cognition of patients. However, it can be hypothesized that those with better performance at a baseline level are more likely to continue the use of CRT even on a remote basis, as engagement with CRT would be better [26].

A novel way found to address the issue of accessibility in the delivery of CRT was by loaning out iPads in a study conducted by Biagianti and colleagues [27]. Though the study was limited in being non-randomized, it provided a unique method of providing CRT to resource-limited settings.

Similarly, Donohoe and colleagues [28] found success in improving neuro-cognition by providing laptops and internet dongles to participants in their study. Additionally, prior training in the use of computers and software being used in the study was provided.

By providing prior guidance in the use of these remote technologies and implementing proper training in the use of the concerned software, dropout rates may decrease and improve accessibility. Additionally, having avenues to provide either the option of a tablet or a laptop for the duration of CRT may enable the usage of CRT as a viable form of improving neurocognitive functionality in resource-limited settings and among vulnerable populations.

## Available research

Table 1 summarizes the current available research in cognitive remediation therapies.

**Table 1:** A summary of the studies done to assess remote cognitive remediation

Authors (year)	Type of study	*N*/group criteria	Intervention	Outcome	Feasibility
Schlosser et al. (2018) [32]	RCT, double blind	PRIME (*n* = 22), Waitlist (*n* = 22)	PRIME	Trust task [71–73] from baseline to post-trial	Retention rate = 74%
Donohoe et al. (2018) [28]	RCT, single blind	CR group (*n* = 48), Control (*n* = 42)	Web-based CR training	Episodic memory, WMS-III [74], SWM from the Cambridge Neurophysiological Test Automated Battery [75]	23 dropouts, 10 lost to follow-up in CR group, 7 drop outs, 13 lost to follow-up in control
Fisher et al. (2015) [69]	RCT, double blind	Auditory training (*n* = 63), computer games (*n* = 58)	Auditory training, computer games	MATRICS [76], symptoms and functioning	19 withdrawals, AT group; 14 withdrawals, CG group
Biagianti et al. (2017) [27]	RCT, double blind, reanalysis of data	Desktop computers (*n* = 33), iPads (*n* = 41)	CRT	MATRICS	Attrition rate = 36%
Medalia et al. (2021) [23]	Mixed methods	Hybrid condition (*n* = 28), all-clinic condition (*n* = 27)	Hybrid CR, CRT in clinic	Qualitative assessment	38 completed subjects (clinic *n* = 18, hybrid *n* = 20)
Loewy et al. (2021) [70]	Intent to treat analysis	Auditory training (*n* = 80), Computer games (*n* = 65)	Auditory training, Computer games	MATRICS, Global Cognition	6-month follow-up completed: AT *n* = 40, CG *n* = 37
Palumbo et al. (2019) [35]	Pilot study	*n* = 8	CIRCuiTS	MCCB [76, 77]	No dropouts

*CIRCuiTS* Computerized Interactive Remediation of Cognition-Training for Schizophrenia, *CR* cognitive remediation, *CRT* cognitive remediation therapy, *MATRICS* Measurement and Treatment Research to Improve Cognition in Schizophrenia, *MCCB* MATRICS Consensus Cognitive Battery, *PRIME* Personalised Real Time Intervention for Motivational Enhancement, *RCT* randomized controlled trial, *SWM* Spatial Working Memory, *WMS-III* Wechsler Memory Scale-3rd edition

Internet and mobile interventions have satisfactory and attainable results in patients diagnosed with schizophrenia and are likely to improve the functional and clinical outcomes [29, 30].

According to the available resources, Granholm [31] and colleagues used mobile devices to administer cognitive behavior therapy (CBT) in a therapeutic context. 55 patients received 840 text messages over a period of 12 weeks targeting medication adherence, socialization and auditory hallucinations.

Text messaging intervention incorporates principles of CBT with four types of message sent for each outcome of interest: outcome and cognitive assessment, pre-elicited thought challenging messages, personalized behavioral coping strategies, significant improvement in auditory hallucinations and socialization. Medication adherence was improved for those living independently, effective in those with higher functioning. Those with lower functioning and more negative symptoms were less likely to complete the intervention [31].

People with recent onset schizophrenia spectrum disorders took part in an RCT of 12 weeks duration to determine efficacy of PRIME [32] (personalized real-time intervention for motivational enhancement), a mobile-based digital health intervention to improve motivation and quality of life. Participants worked towards self-identified goals with the support of a virtual community of age-matched patients with schizophrenia spectrum disorder as well as motivation. There was a significant improvement in depression, defeatist beliefs, self-efficacy. Motivation and negative symptoms were improved post-trial and these improvements were maintained 3 months after the end of the trial.

The other form of digital therapy is SlowMo [33] app, which is a digital therapy to target fear of harm from others. It aims to adopt interventionist casual treatment approach; it mainly tackles fast thinking, which has a key role in development and maintenance of distressing paranoia. Incorporating digital technology into psychological therapy helps in effective and accessible treatment.

CR given in hybrid approach as studied by Medalia and colleagues [23] have both feasibility and acceptability as it allows half the sessions to be conducted remotely as homework. Due to limited access to technology, participants had to come to clinic for guidance of the clinician to finish the homework.

Another study of single blind Randomized Controlled Trial (RCT) of CRT, which specifically targeted on working memory was a study conducted by Donohoe and colleagues [28]. Here individuals received only 30–60 min of support per week from the therapist showed significant benefits in memory performance compared with treatment as usual. Prior to the commencement of the training program, computer access and training needs were evaluated.

Evidence further suggests that CRT can be easily delivered through mobile platforms. Four-week trial delivery of cognitive training through ipad versus treatment as usual to 20 patients diagnosed with first episode schizophrenia had good improvement in working memory, better adherence and acceptability rate [34]. It is feasible and acceptable to engage patients diagnosed with schizophrenia in training of social cognition completely by remote ipad use [27]. Supplementary cognitive training along with social cognition exercise as compared to cognitive training alone, there was no significant difference between ipad and desktop version of cognitive training in terms of the stimulus sets, stimulus progressions, adapting parameters of each exercise.

Computerized Interactive Remediation of Cognition-Training for Schizophrenia (CIRCuiTS) [35] is a pilot study that implements CRT using computer software. It is developed to target neurocognitive, metacognitive deficits of people diagnosed with schizophrenia. Implemented in Italian, patients were assessed at baseline and went on to receive forty CIRCuiTS therapy sessions, three times a week for an hour over a 3-month period. Post-treatment, participants were reassessed. It had high feasibility and good acceptability in terms of number of dropouts and patient’s schizophrenia. Patient improved in learning, speed of processing and working memory. Additionally, there was a reduction in disorganization.

## Challenges

The research that has been conducted has predominantly stemmed from higher income countries. Considerations such as ability to translate the successes of the research into real-world settings are important. Some of the potential challenges are:

Patients’ cognitive deficits may cause a limitation in ability to purchase and set up equipment: although some studies have quoted the use of assistive technology in patients with schizophrenia [36, 37], the cognitive deficits caused by the disease may provide an impediment in the likelihood of adoption of technology. In patients with significant deficits, accessibility and purchase power may be a limiting factor [38, 39], impeding the applicability of cognitive remediation in real-world settings.

Secondly, privacy of the individual may not be maintained in the remote access of cognitive training: privacy concerns are common with the increasing use of technology in healthcare [40]. While privacy laws are in place in certain countries to guide telehealth practices, many nations are still in the transition of digitization of healthcare data [41–44]. Legal instruments need to be developed prior to the widespread usage of remote interventions that will effectively protect data and prevent misuse.

Another consideration is that the cost of devices used for the remote access of cognitive training may be more than that of the cognitive training delivered in person [45]. Cost-effectiveness studies may guide future recommendations in this regard and help elucidate the differences that may present during in-person therapy as opposed to remote CR. Another likely problem to be encountered is glitches in Internet connections. Internet connectivity favors urban populations disproportionately [46, 47]. CR has shown improvements with sustained therapy. While remote CR may be useful in improving accessibility to people living in rural areas, there are certainly practical limitations.

If training workforce is insufficient then the delivery of the remote access may not be standardized. With the recent pandemic, medical education has taken a turn towards the virtual space [48, 49]. Tele-health curriculum has been integrated as key parts of residency training and in nursing education [50–53]. However, much of these efforts are still in the phase of capacity building. Training an effective workforce to deliver CR remotely is still a challenge in the present scenario.

Online training may hamper the ability to have meaningful face-to-face encounters with patients. Social isolation, a central issue in schizophrenia [54], may rise in the absence of in-person interventions. A discomfort among both service users and providers with these treatment modalities may be anticipated. The transitions and adaptation to remote administration as either the sole or primary encounter type may then prove to be difficult. Audio only delivery may pose challenges with some clients, as body language and mood shifts are difficult to assess [55–57]. Use of technological devices in smart phone could actually fuel delusions as they may think that someone is controlling by outside force.

## Advantages

- Internet interventions focused have improved clinical engagement in patients with schizophrenia [58]. Text messages and smartphone apps have shown an improvement in health literacy among patients, better self-management and further, served to increase pharmacological adherence [59–63]. Additionally, widespread availability, internet connectivity, portability and ease of use of mobile-based interventions are both acceptable and feasible to be used for patients diagnosed with schizophrenia.
- Mobile-based technologies are nowadays accessible to socially and economically disadvantaged people [64, 65]. This paves the way for better health equity and inclusivity [66]. The ability to access technology in developing parts of the world proves to be an impetus to drive research efforts towards tele-psychiatry in the goal to improve access. In addition to improving health equity, remote interventions also serve to provide patients with more privacy. For instance, young adults would prefer to communicate using computers and cell phones rather than approach therapists in person [67]. Some of the stigma associated with seeking mental health interventions is reduced in the face of more privacy using video-calling features that are available in app-based mental health services [68].
- Remote therapy helps overcoming difficulties associated with social interaction in individuals diagnosed with schizophrenia. Along with that, the disinhibiting effect of online communication and potential for such communication to overcome the fear of stigma by removing face-to-face interaction has been seen. So in therapy, this disinhibition can enhance therapeutic expression and self-reflection by helping patients to be more open about their illness.

## Feasibility of remote cognitive remediation therapy

The use of remote interventions in the delivery of CRT has been encouraging. While there are some barriers, the CRT apps and those provided by different modalities have shown a fair engagement.

Schlosser and colleagues [32] implemented CR interventions using the PRIME (Personalised Real Time Intervention for Motivational Enhancement) app. This app provided participants with a peer community; cognitive behavior therapy (CBT) based coaching and promoted goal-directed behavior using social reinforcement. The app recorded a high degree of engagement with about 5152 direct messages sent by participants to their coaches and with a login rate of over 4 days/week. The retention rate recorded in this study was 74% with it rising to 88% post-intervention.

Donohue and colleagues [28] utilized online CR training programs in their study. However, they noted a higher dropout rate compared to Schlosser et al., especially for their intervention group. The intervention group had a retention rate of only 31.25%. The dropout from the study was greatest in the first 1–2 weeks. This suggests that efforts need to be directed in the initial period of starting any remote therapy towards identifying factors that may lead to patients having difficulty in using these remote interventions.

Fisher and colleagues [69] have found considerable success in improving global cognition, verbal memory and problem solving using auditory training. This group had about 40 out of 63 patients complete the required 20–40 h of auditory training imparted using laptops.

Biagianti and colleagues [27] conducted CRT loaning out tablets and compared this group with a group who were delivered CRT in a lab on desktop computers. The attrition rate was 36.6% with no significant difference between the two groups in attrition or adherence. This study found it feasible to impart CRT using tablets and had similar cognitive and functional outcomes between patients who were delivered to CRT in a laboratory and those who received it at home on iPads. This study, however, had a small sample size and was not randomized. Much of the users of iPad were younger in age range.

Medalia [23] compared clinician-led clinic CRT with hybrid CR in which patients were assigned “homework sheets” and asked to complete CRT on internet connected tablets or computers. Retention rates were similar among the two groups (clinic = 72%, hybrid = 76.9%, *p* = 0.755; adjusted for COVID 19). The perceived benefit of this modality was positive among the subjects.

Loewy [70] found improvements in global cognition; problem solving and speed of processing in patients who underwent remotely completed intensive auditory training. Though attrition rates at 6 months follow-up approached 50%, they did find a considerable improvement in overall cognition in patients who did complete the 20–40 h of auditory training.

The findings of the study conducted by Palumbo and colleagues [35] using the Computerized Interactive Remediation of Cognition-Training for Schizophrenia (CIRCuiTS) found no dropouts. Though there were uniform improvements in cognition among those who received the intervention, it was limited by a small sample size (*n* = 8) and likely may have attrition on scaling up.

Besides these studies, there are other trials that are ongoing assessing the accessibility and feasibility of remote CRT interventions. The SlowMo [33] is likely undergoing trials for an assessment of its efficacy, accessibility and feasibility in a clinical setting for patients with paranoia.

## Limitations and future directions

The present studies have shown preliminary benefits in improving cognitive functioning through remote interventions and can be feasible and accessible to schizophrenia patients. However, the current available research in remote CR is limited by small sample sizes and high attrition rates. For widespread implementation of these modalities, more robust methodology needs to be adopted in controlled trial settings with a much greater sample size. Use of smartphone apps, for instance, may be helpful in conducting such studies with a greater retention of study participants and provide data in the true therapeutic benefits that remote therapies may be able to confer.

Another aspect that needs to be addressed is the demographics of the present study. These studies have taken place in developed nations. Rigorous studies in developing countries may be effective in providing a better picture of implementation in underserved and rural areas. Future research in this area may help in creating infrastructure towards improving accessibility of remote CR in areas typically where it may be inaccessible.

Lastly, comparison studies may be useful in comparing various modes of implementation of remote CR. For example, comparing in-person control arm with desktop, tablet and smartphone application may provide insight into technologies that may provide the patient with the greatest benefits. A hybrid model may also be used to compare efficacies and expand on our current understanding of the efficacy of these remote interventions.

## Conclusions

While in-person therapy cannot be substituted, remote interventions using mobile phones, tablets and laptops provide a good alternative in resource-limited settings and augment mental health service where in-person therapy is indeed available. A hybrid approach may be the best option in these times.

It is important to consider the necessity of these CR measures beyond treating the positive symptoms of schizophrenia to improve the functionality of patients. With CRT being delivered remotely, it provides the ability for mental health care providers to routinely check in on patients, assess progress in CR, and supplement with “booster sessions” should the need arise in follow-up sessions. It might be more effective in younger patients, with lesser cognitive impairment and with a higher level of functioning as a way to supplement rather than reverse the cognitive impairment.

Leveraging tele-psychiatry sessions in the context of schizophrenia can have profound improvements in the way disability in schizophrenia is present. The rehabilitation of schizophrenic patients socially and economically is a challenge that has been facing the mental health community for a long time. Using remote CRT measures provides a bridge towards that goal of successful rehabilitation and reintegration.

## Data Availability

All data generated or analyzed in this article are available in the referenced publications.

## Abbreviations

CBT: Cognitive behavior therapy
CIRCuiTS: Computerized Interactive Remediation of Cognition-Training for Schizophrenia
CR: Cognitive remediation
CRT: Cognitive remediation therapy
MATRICS: Measurement and Treatment Research to Improve Cognition in Schizophrenia
MCCB: MATRICS Consensus Cognitive Battery
PRIME: Personalised Real Time Intervention for Motivational Enhancement
RCT: Randomized controlled trial
SWM: Spatial Working Memory
WMS-III: Wechsler Memory Scale-3rd edition

## References

[1] Silberstein J, Harvey PD (2019). Cognition, social cognition, and Self-assessment in schizophrenia: prediction of different elements of everyday functional outcomes. CNS Spectr.

[2] McGurk SR, Twamley EW, Sitzer DI, McHugo GJ, Mueser KT (2007). A meta-analysis of cognitive remediation in schizophrenia. Am J Psychiatry.

[3] Radfar A, Ferreira MM, Sosa JP, Filip I (2021). Emergent crisis of COVID-19 pandemic: mental health challenges and opportunities. Front Psychiatry.

[4] Barlati S, Deste G, De Peri L, Ariu C, Vita A (2013). Cognitive remediation in schizophrenia: current status and future perspectives. Schizophr Res Treat.

[5] Wykes T, Reeder C, Landau S, Everitt B, Knapp M, Patel A (2007). Cognitive remediation therapy in schizophrenia: randomised controlled trial. Br J Psychiatry.

[6] McGurk SR, Mueser KT, DeRosa TJ, Wolfe R (2009). Work, recovery, and comorbidity in schizophrenia: a randomized controlled trial of cognitive remediation. Schizophr Bull.

[7] Hodge MA, Siciliano D, Withey P, Moss B, Moore G, Judd G (2010). A randomized controlled trial of cognitive remediation in schizophrenia. Schizophr Bull.

[8] van der Gaag M, Kern RS, van den Bosch RJ, Liberman RP (2002). A controlled trial of cognitive remediation in schizophrenia. Schizophr Bull.

[9] Revell ER, Neill JC, Harte M, Khan Z, Drake RJ (2015). A systematic review and meta-analysis of cognitive remediation in early schizophrenia. Schizophr Res.

[10] Eack SM, Hogarty GE, Cho RY, Prasad KM, Greenwald DP, Hogarty SS (2010). Neuroprotective effects of cognitive enhancement therapy against gray matter loss in early schizophrenia: results from a 2-year randomized controlled trial. Arch Gen Psychiatry.

[11] Griffiths L, Blignault I, Yellowlees P (2006). Telemedicine as a means of delivering cognitive-behavioural therapy to rural and remote mental health clients. J Telemed Telecare.

[12] Kasckow J, Felmet K, Appelt C, Thompson R, Rotondi A, Haas G (2014). Telepsychiatry in the assessment and treatment of schizophrenia. Clin Schizophr Relat Psychoses.

[13] Ben-Zeev D, Brenner CJ, Begale M, Duffecy J, Mohr DC, Mueser KT (2014). Feasibility, acceptability, and preliminary efficacy of a smartphone intervention for schizophrenia. Schizophr Bull.

[14] Naslund JA, Aschbrenner KA, Bartels SJ (2016). Wearable devices and smartphones for activity tracking among people with serious mental illness. Ment Health Phys Act.

[15] Gottlieb JD, Romeo KH, Penn DL, Mueser KT, Chiko BP (2013). Web-based cognitive-behavioral therapy for auditory hallucinations in persons with psychosis: a pilot study. Schizophr Res.

[16] Freeman D, Reeve S, Robinson A, Ehlers A, Clark D, Spanlang B (2017). Virtual reality in the assessment, understanding, and treatment of mental health disorders. Psychol Med.

[17] Cuijpers P, van Straten A, Andersson G (2008). Internet-administered cognitive behavior therapy for health problems: a systematic review. J Behav Med.

[18] Álvarez-Jiménez M, Gleeson JF, Bendall S, Lederman R, Wadley G, Killackey E (2012). Internet-based interventions for psychosis: a sneak-peek into the future. Psychiatr Clin N Am.

[19] Gaebel W, Großimlinghaus I, Kerst A, Cohen Y, Hinsche-Böckenholt A, Johnson B (2016). European Psychiatric Association (EPA) guidance on the quality of eMental health interventions in the treatment of psychotic disorders. Eur Arch Psychiatry Clin Neurosci.

[20] Eberly LA, Kallan MJ, Julien HM, Haynes N, Khatana SA, Nathan AS (2020). Patient characteristics associated with telemedicine access for primary and specialty ambulatory care during the COVID-19 pandemic. JAMA Netw Open.

[21] Torous J, Keshavan M (2020). COVID-19, mobile health and serious mental illness. Schizophr Res.

[22] Gruber J, Prinstein MJ, Clark LA, Rottenberg J, Abramowitz JS, Albano AM (2021). Mental health and clinical psychological science in the time of COVID-19: challenges, opportunities, and a call to action. Am Psychol.

[23] Medalia A, Saperstein AM, Stefancic A, Meyler S, Styke S, Qian M (2021). Feasibility and acceptability of remotely accessed cognitive remediation for schizophrenia in public health settings. Psychiatry Res.

[24] Zhang D, Shi L, Han X, Li Y, Jalajel NA, Patel S (2021). Disparities in telehealth utilization during the COVID-19 pandemic: Findings from a nationally representative survey in the United States. J Telemed Telecare.

[25] Ng BP, Park C (2021). Peer reviewed: accessibility of telehealth services during the COVID-19 pandemic: a cross-sectional survey of medicare beneficiaries. Prev Chronic Dis.

[26] Biagianti B, Fisher M, Neilands TB, Loewy R, Vinogradov S (2016). Engagement with the auditory processing system during targeted auditory cognitive training mediates changes in cognitive outcomes in individuals with schizophrenia. Neuropsychology.

[27] Biagianti B, Fisher M, Howard L, Rowlands A, Vinogradov S, Woolley J (2017). Feasibility and preliminary efficacy of remotely delivering cognitive training to people with schizophrenia using tablets. Schizophr Res Cogn.

[28] Donohoe G, Dillon R, Hargreaves A, Mothersill O, Castorina M, Furey E (2018). Effectiveness of a low support, remotely accessible, cognitive remediation training programme for chronic psychosis: cognitive, functional and cortical outcomes from a single blind randomised controlled trial. Psychol Med.

[29] Alvarez-Jimenez M, Alcazar-Corcoles MA, González-Blanch C, Bendall S, McGorry PD, Gleeson JF (2014). Online, social media and mobile technologies for psychosis treatment: a systematic review on novel user-led interventions. Schizophr Res.

[30] Biagianti B, Hidalgo-Mazzei D, Meyer N (2017). Developing digital interventions for people living with serious mental illness: perspectives from three mHealth studies. Evid Based Ment Health.

[31] Granholm E, Ben-Zeev D, Link PC, Bradshaw KR, Holden JL (2012). Mobile Assessment and Treatment for Schizophrenia (MATS): a pilot trial of an interactive text-messaging intervention for medication adherence, socialization, and auditory hallucinations. Schizophr Bull.

[32] Schlosser DA, Campellone TR, Truong B, Etter K, Vergani S, Komaiko K (2018). Efficacy of PRIME, a mobile app intervention designed to improve motivation in young people with schizophrenia. Schizophr Bull.

[33] Garety PA, Ward T, Freeman D, Fowler D, Emsley R, Dunn G (2017). SlowMo, a digital therapy targeting reasoning in paranoia, versus treatment as usual in the treatment of people who fear harm from others: study protocol for a randomised controlled trial. Trials.

[34] Dang J, Zhang J, Guo Z, Lu W, Cai J, Shi Z (2014). A pilot study of iPad-assisted cognitive training for schizophrenia. Arch Psychiatr Nurs.

[35] Palumbo D, Mucci A, Giordano GM, Piegari G, Aiello C, Pietrafesa D (2019). The efficacy, feasibility and acceptability of a remotely accessible use of CIRCuiTS, a computerized cognitive remediation therapy program for schizophrenia: a pilot study. Neuropsychiatr Dis Treat.

[36] Gay K, Torous J, Joseph A, Pandya A, Duckworth K (2016). Digital technology use among individuals with schizophrenia: results of an online survey. JMIR Ment Health.

[37] Devlin H, Nolan C, Turner N (2019). Assistive technology and schizophrenia. Ir J Occup Ther.

[38] Lien WC, Wang WM, Wang HD, Lin FH, Yao FZ (2021). Environmental barriers and functional outcomes in patients with Schizophrenia in Taiwan: the capacity-performance discrepancy. Int J Environ Res Public Health.

[39] Wong KT, Liu D, Balzan R, King D, Galletly C (2020). Smartphone and internet access and utilization by people with schizophrenia in South Australia: quantitative survey study. JMIR Ment Health.

[40] Hale TM, Kvedar JC (2014). Privacy and security concerns in telehealth. Virtual Mentor.

[41] Baker DC, Bufka LF (2011). Preparing for the telehealth world: navigating legal, regulatory, reimbursement, and ethical issues in an electronic age. Prof Psychol Res Pract.

[42] Cortelyou-Ward K, Atkins DN, Noblin A, Rotarius T, White P, Carey C (2020). Navigating the digital divide: barriers to telehealth in rural areas. J Health Care Poor Underserved.

[43] Brous E (2016). Legal considerations in telehealth and telemedicine. Am J Nurs.

[44] Gajarawala SN, Pelkowski JN (2021). Telehealth benefits and barriers. J Nurse Pract.

[45] Naslund JA, Mitchell LM, Joshi U, Nagda D, Lu C (2022). Economic evaluation and costs of telepsychiatry programmes: a systematic review. J Telemed Telecare.

[46] Strover S (2001). Rural internet connectivity. Telecomm Policy.

[47] Whitacre BE, Mills BF (2010). A need for speed? Rural Internet connectivity and the no access/dial-up/high-speed decision. Appl Econ.

[48] Wilcha RJ (2020). Effectiveness of virtual medical teaching during the COVID-19 crisis: systematic review. JMIR Med Educ.

[49] Tabatabai S (2020). COVID-19 impact and virtual medical education. J Adv Med Educ Prof.

[50] Hoffman P, Kane JM (2015). Telepsychiatry education and curriculum development in residency training. Acad Psychiatry.

[51] Saeed SA, Johnson TL, Bagga M, Glass O (2017). Training residents in the use of telepsychiatry: review of the literature and a proposed elective. Psychiatr Q.

[52] Crawford A, Sunderji N, López J, Soklaridis S (2016). Defining competencies for the practice of telepsychiatry through an assessment of resident learning needs. BMC Med Educ.

[53] Rutledge CM, O’Rourke J, Mason AM, Chike-Harris K, Behnke L, Melhado L (2021). Telehealth competencies for nursing education and practice: the four P’s of telehealth. Nurse Educ.

[54] Kohn ML, Clausen JA (1955). Social isolation and schizophrenia. Am Sociol Rev.

[55] Vaskinn A, Sundet K, Østefjells T, Nymo K, Melle I, Ueland T (2016). Reading emotions from body movement: a generalized impairment in schizophrenia. Front Psychol.

[56] Egeland J, Holmen TL, Bang-Kittilsen G, Bigseth TT, Vaskinn A, Engh JA (2019). Body language reading of emotion and outcome in schizophrenia. Cogn Neuropsychiatry.

[57] Engelstad KN, Sundet KS, Andreassen OA, Vaskinn A (2017). Body language reading of emotion in schizophrenia: associations with symptoms and functional outcome. Scand J Psychol.

[58] Firth J, Torous J (2015). Smartphone apps for schizophrenia: a systematic review. JMIR Mhealth Uhealth.

[59] Miller BJ, Stewart A, Schrimsher J, Peeples D, Buckley PF (2015). How connected are people with schizophrenia? Cell phone, computer, email, and social media use. Psychiatry Res.

[60] Firth J, Cotter J, Torous J, Bucci S, Firth JA, Yung AR (2016). Mobile phone ownership and endorsement of “mHealth” among people with psychosis: a meta-analysis of cross-sectional studies. Schizophr Bull.

[61] Naslund JA, Marsch LA, McHugo GJ, Bartels SJ (2015). Emerging mHealth and eHealth interventions for serious mental illness: a review of the literature. J Ment Health.

[62] D’Arcey J, Collaton J, Kozloff N, Voineskos AN, Kidd SA, Foussias G (2020). The use of text messaging to improve clinical engagement for individuals with psychosis: systematic review. JMIR Ment Health.

[63] Curto M, Fazio F, Ulivieri M, Navari S, Lionetto L, Baldessarini RJ (2021). Improving adherence to pharmacological treatment for schizophrenia: a systematic assessment. Expert Opin Pharmacother.

[64] Chukwuere JE. Social media accessibility: opportunities and challenges for rural communities. In: International conference on emerging technology and interdisciplinary sciences 2021, pp. 2–8.

[65] Narasimhan N. Digital accessibility in the Asia-Pacific Region. In: Accessible technology and the developing world. 2021:106. 10.1093/oso/9780198846413.003.0006.

[66] Friis-Healy EA, Nagy GA, Kollins SH (2021). It is time to REACT: opportunities for digital mental health apps to reduce mental health disparities in racially and ethnically minoritized groups. JMIR Ment Health.

[67] Melcher J, Camacho E, Lagan S, Torous J (2022). College student engagement with mental health apps: analysis of barriers to sustained use. J Am Coll Health.

[68] Torous J, Bucci S, Bell IH, Kessing LV, Faurholt-Jepsen M, Whelan P (2021). The growing field of digital psychiatry: current evidence and the future of apps, social media, chatbots, and virtual reality. World Psychiatry.

[69] Fisher M, Loewy R, Carter C, Lee A, Ragland JD, Niendam T (2015). Neuroplasticity-based auditory training via laptop computer improves cognition in young individuals with recent onset schizophrenia. Schizophr Bull.

[70] Loewy R, Fisher M, Ma S, Carter C, Ragland JD, Niendam TA (2021). Durable cognitive gains and symptom improvement are observed in individuals with recent-onset schizophrenia 6 months after a randomized trial of auditory training completed remotely. Schizophr Bull.

[71] Campellone TR, Kring AM (2018). Anticipated pleasure for positive and negative social interaction outcomes in schizophrenia. Psychiatry Res.

[72] Campellone TR, Fisher AJ, Kring AM (2016). Using social outcomes to inform decision-making in schizophrenia: relationships with symptoms and functioning. J Abnorm Psychol.

[73] Campellone TR, Truong B, Gard D, Schlosser DA (2018). Social motivation in people with recent-onset schizophrenia spectrum disorders. J Psychiatr Res.

[74] Wechsler D (1997). Wechsler Memory Scale (WMS–III).

[75] Robbins TW, James M, Owen AM, Sahakian BJ, McInnes L, Rabbitt P (1994). Cambridge Neuropsychological Test Automated Battery (CANTAB): a factor analytic study of a large sample of normal elderly volunteers. Dementia.

[76] Nuechterlein KH, Green MF, Kern RS, Baade LE, Barch DM, Cohen JD (2008). The MATRICS Consensus Cognitive Battery, part 1: test selection, reliability, and validity. Am J Psychiatry.

[77] Kern RS, Nuechterlein KH, Green MF, Baade LE, Fenton WS, Gold JM (2008). The MATRICS Consensus Cognitive Battery, part 2: co-norming and standardization. Am J Psychiatry.

